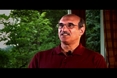# The 2009 Lindau Nobel Laureate Meeting: Martin Chalfie, Chemistry 2008

**DOI:** 10.3791/1570

**Published:** 2010-02-10

**Authors:** Martin Chalfie

## Abstract

American Biologist Martin Chalfie shared the 2008 Nobel Prize in Chemistry with Roger Tsien and Osamu Shimomura for their discovery and development of the Green Fluorescent Protein (GFP).

Martin Chalfie was born in Chicago in 1947 and grew up in Skokie Illinois.  Although he had an interest in science from a young age-- learning the names of the planets and reading books about dinosaurs-- his journey to a career in biological science was circuitous.  In high school, Chalfie enjoyed his AP Chemistry course, but his other science courses did not make much of an impression on him, and he began his undergraduate studies at Harvard uncertain of what he wanted to study.  Eventually he did choose to major in Biochemistry, and during the summer between his sophomore and junior years, he joined Klaus Weber's lab and began his first real research project, studying the active site of the enzyme aspartate transcarbamylase. Unfortunately, none of the experiments he performed in Weber's lab worked, and Chalfie came to the conclusion that research was not for him.

Following graduation in 1969, he was hired as a teacher Hamden Hall Country Day School in Connecticut where he taught high school chemistry, algebra, and social sciences for 2 years.  After his first year of teaching, he decided to give research another try.  He took a summer job in Jose Zadunaisky's lab at Yale, studying chloride transport in the frog retina.  Chalfie enjoyed this experience a great deal, and having gained confidence in his own scientific abilities, he applied to graduate school at Harvard, where he joined the Physiology department in 1972 and studied norepinephrine synthesis and secretion under Bob Pearlman.  His interest in working on *C. elegans* led him to post doc with Sydney Brenner, at the Medical Research Council Laboratory of Molecular Biology in Cambridge, England.  In 1982 he was offered position at Columbia University.

When Chalfie first heard about GFP at a research seminar given by Paul Brehm in 1989, his lab was studying genes involved in the development and function of touch-sensitive cells in *C. elegans*.  He immediately became very excited about the idea of expressing the fluorescent protein in the nematode, hoping to figure out where the genes were expressed in the live organism.  At the time, all methods of examining localization, such as antibody staining or *in situ* hybridization, required fixation of the tissue or cells, revealing the location of proteins only at fixed points in time.

In September 1992, after obtaining GFP DNA from Douglas Prasher, Chalfie asked his rotation student, Ghia Euskirchen to express GFP in *E. coli*, unaware that several other labs were also trying to express the protein, without success. Chalfie and Euskirchen used PCR to amplify only the coding sequence of GFP, which they placed in an expression vector and expressed in *E.coli*.  Because of her engineering background, Euskirchen knew that the microscope in the Chalfie lab was not good enough to use for this type of experiment, so she captured images of green bacteria using the microscope from her former engineering lab.  This work demonstrated that GFP fluorescence requires no component other than GFP itself.  In fact, the difficulty that other labs had encountered stemmed from their use of restriction enzyme digestions for subcloning, which brought along an extra sequence that prevented GFP's fluorescent expression. Following Euskirchen's successful expression in* E. coli*, Chalfie's technician Yuan Tu went on to express GFP in *C. elegans*, and Chalfie published the findings in Science in 1994.

Through the study of *C. elegans* and GFP, Chalfie feels there is an important lesson to be learned about the importance basic research.  Though there has been a recent push for clinically-relevant or patent-producing (translational) research, Chalfie warns that taking this approach alone is a mistake, given how "woefully little" we know about biology.   He points out the vast expanse of the unknowns in biology, noting that important discoveries such as GFP are very frequently made through basic research using a diverse set of model organisms. Indeed, the study of GFP bioluminescence did not originally have a direct application to human health. Our understanding of it, however, has led to a wide array of clinically-relevant discoveries and developments. Chalfie believes we should not limit ourselves:  "We should be a little freer and investigate things in different directions, and be a little bit awed by what we're going to find."

**Figure:**